# Tau oligomers accumulation sensitizes prostate cancer cells to docetaxel treatment

**DOI:** 10.1007/s00432-021-03598-3

**Published:** 2021-04-02

**Authors:** Stefano Martellucci, Letizia Clementi, Samantha Sabetta, Paola Muzi, Vincenzo Mattei, Mauro Bologna, Adriano Angelucci

**Affiliations:** 1 Department of Biotechnological and Applied Clinical Sciences, University of L’Aquila, Via Vetoio, 67100 L’Aquila, Italy; 2 Biomedicine and Advanced Technologies Rieti Center “Sabina Universitas”, 02100 Rieti, Italy; 3 Department of Life, Health and Environmental Sciences, University of L’Aquila, 67100 L’Aquila, Italy; 4 Department of Experimental Medicine, “Sapienza” University, 00161 Rome, Italy

**Keywords:** Tau oligomer, Autophagy, Prostate Cancer, Docetaxel, Cancer Therapy, Mitosis

## Abstract

**Purpose:**

Human tau is a highly dynamic, multifunctional protein expressed in different isoforms and conformers, known to modulate microtubule turnover. Tau oligomers are considered pathologic forms of the protein able to initiate specific protein accumulation diseases, called tauopathies. In our study, we investigated the potential association between autophagy and tau oligomers accumulation and its role in the response of prostate cancer cells to docetaxel.

**Methods:**

We evaluated in vitro the expression of tau oligomers in prostate cancer cell lines, PC3 and DU145, in presence of autophagy inhibitors and investigated the role of tau oligomers accumulation in resistance to docetaxel treatment.

**Results:**

Tau protein was basally expressed in prostate cancer lines as several monomeric and oligomeric forms. The pharmacologic inhibition of autophagy induced in cancer cells the accumulation of tau protein, with a prevalent expression of oligomeric forms. Immunofluorescence analysis of untreated cells revealed that tau was visible mainly in dividing cells where it was localized on the mitotic spindle. Inhibition of autophagy determined an evident upregulation of tau signal in dividing cells and the presence of aberrant monoastral mitotic spindles. The accumulation of tau oligomers was associated with DNA DSB and increased cytotoxic effect by docetaxel.

**Conclusions:**

Our data indicate that autophagy could exert a promoting role in cancer growth and during chemotherapy facilitating degradation of tau protein and thus blocking the antimitotic effect of accumulated tau oligomers. Thus, therapeutic strategies aimed at stimulating tau oligomers formation, such as autophagy inhibition, could be an effective adjuvant in cancer therapy.

## Introduction

Tau is a heat-stable, highly dynamic, protein known to bind to and stabilize microtubules (Wang and Mandelkow 2016; Kellogg et al. 2018). Tau exists in different isoforms and folds with specific conformers which are considered the etiologic factors of protein accumulation diseases collectively known as tauopathies (Fitzpatrick et al. 2017). Besides the six splice variants generated from a single gene through alternative splicing of exons 2, 3 and 10 (0, 1, 2 N multiplied by C-terminal microtubule-binding repeats 3R and 4R), and three major conformations described, phosphorylation and other post-translational modifications contribute to generate a very complicated array of protein expression.

Tau accumulation into intracellular tangles has long been associated with neurodegenerative disease, including Alzheimer’s disease, Parkinson’s disease, progressive supranuclear palsy, corticobasal degeneration and frontotemporal dementias. More recently new data are unveiling new physiopathologic aspects of tau. For example, it was demonstrated that also non-fibrillar soluble aggregates and oligomers of tau have a pathogenic activity (Lasagna-Reeves et al. 2012). In fact, soluble oligomers can self-aggregate to form large insoluble toxic fibrils, playing a crucial role for neurodegenerative diseases onset and acting as seeds in the misfolding and the propagation of the disease. In particular, hyperphosphorylated tau tends to be spontaneously misfolded and rapidly aggregated into oligomers enriched in β-sheet content (Ward et al. 2012). At the same time, available evidence suggests that the accumulation of the smaller, soluble and dynamic oligomers is also associated with cellular stress and mitochondria dysfunction (Shafiei et al. 2017). Treatment with curcumin derivatives able to counteract tau oligomer aggregation pathways, protected neuroblastoma SH-SY5Y cells from tau oligomer-induced toxicity, suggesting that the aggregation state of toxic tau oligomeric species could be an effective therapeutic target (Lo Cascio et al. 2019). In agreement, the use of specific antibodies able to reduce levels of tau oligomers has been effectively used in the therapeutic protection against tau pathologies in vivo (Castillo-Carranza et al. 2015).

The cytotoxicity associated with tau accumulation prompts to investigate the importance of physiologic clearance mechanism in tau homeostasis. Indeed, increasing data are indicating a protective role for protein clearance mechanisms in different cell models and not only in neurodegenerative diseases. Protein quality control via ubiquitin/proteasome system and autophagy are particularly important for the timely removal of aggregated forms of pathogenic proteins, including tau (Ciechanover and Kwon 2015). Dysregulation of autophagy is frequently seen in tauopathies, and some authors indicate in this situation a driving pathogenic role (Menzies et al. 2017; Silva et al. 2019). Hyperphosphorylated tau co-localizes with autophagic vesicles in patient with tauopathies and increased levels of the lysosomal components were reported in the same patients, indicating the activation of a recovery mechanism (Piras et al. 2016).

In general, autophagy is increasingly considered an important mechanism in the maintenance of cellular homeostasis. In fact, beside serving as a stress response in presence of increased energy or structural demand, autophagic activity serves to dispose of unwanted organelles or proteins or during normal cell metabolism. In fact, autophagy targets include aberrant mitochondria but also several protein aggregates (Kaur and Debnath 2015).

The hypothesis that tau was expressed prevalently in neurons justifies why tau biology has been investigated almost only in the nervous central system. Indeed, tauopathies are described as neurodegenerative diseases. However, tau role is increasingly attracting attention also in other pathologies, including non-neuronal cancers. Tau expression in cancer cell lines and tissues is highly heterogeneous, with negative cases frequently reported. The principal human tissues for which has been reported a measurable level of tau expression are breast, prostate, gastric, colorectal pancreatic tissues and the same for corresponding tumors (Li et al. 2013; Huda et al. 2017; Wang et al. 2013; Jimeno et al. 2007; Souter and Lee 2009; Gargini et al. 2019). Although the significance of high expression heterogeneity also in these tissues is unknown, some data suggest a potential association with cancer progression and prognosis. In fact, tau is an independent prognostic marker in prostate cancer and tau expression was increased in metastatic tissue compared with primary breast cancer cells (Schroeder et al. 2019; Matrone et al. 2010). In addition, tau expression in cancer cells can predict response to chemotherapy, and in particular to taxanes. Tau-negative expression has been repetitively related to a favourable response to paclitaxel treatment in the breast, ovarian gastric and bladder cancer (Rouzier et al. 2005; Smoter et al. 2013; Mimori et al. 2006; Wosnitzer et al. 2011). In addition, other authors, after observing a higher cancer incidence in families affected by mutated tau and tauopathies, indicated in dysfunctional tau a novel risk factor for cancer (Rossi et al. 2018).

These initial suggestions recommend further investigation about the possibility that tau expression could represent an advantage for cancer progression in some contexts, and that tau homeostasis could be a therapeutic target. Starting from this hypothesis we evaluated the expression of tau in prostate cancer cell lines and the role of tau oligomers accumulation in cells treated with docetaxel.

## Materials and methods

### Cell lines

PC3, a human prostate carcinoma cell line established from bone metastasis (ECACC, #90112714), was cultured in Coon’s Modified Ham’s medium supplemented with 10% fetal bovine serum, 2 mM glutamine 100 IU/mL penicillin and 100 μg/mL streptomycin. DU145 a human prostate carcinoma cell line established from brain metastasis (ATCC, HTB-81) was cultured in Eagle’s Minimum Essential Medium (EMEM) supplemented with 10% fetal bovine serum, 2 mM glutamine 100 IU/mL penicillin and 100 μg/mL streptomycin. All cell lines underwent testing for mycoplasma by culture isolation, Hoechst DNA staining and PCR, together with culture testing for contaminant bacteria, yeast and fungi. Authentication procedures included species verification by DNA barcoding and identity verification by DNA profiling. Human cell lines were analyzed by PCR of short tandem repeat sequences within chromosomal microsatellite DNA (STR-PCR). Cells were plated at a density of 10^4^ cells/cm^2^, incubated in 5% CO_2_ at 37 °C and recovered after different times of incubation. At the endpoint, cells were harvested, centrifuged and aliquots of cell suspensions were counted using a Neubauer hemocytometer chamber. Dead cells were assessed by the trypan blue dye (Sigma-Aldrich, St. Louis, MO, USA) exclusion test. Authophagy inhibitors, chloroquine and bafilomycin A1, and inhibitor of tau oligomers, methylene blue were purchased from Sigma-Aldrich.

### Western blot

Cancer cells were processed for protein extraction with cell lysis buffer containing 0.1% Triton X-100, 10 mM Tris–HCl (pH 7.5), 150 mM NaCl, 5 mM EDTA, and supplemented with 1 mM Na_3_VO_4_ and 75 U of aprotinin (Sigma-Aldrich), and incubated for 20 min at 4 °C. The cell lysate was centrifuged for 10 min at 1300 g to eliminate nuclei and large cellular debris. After protein concentration analysis by Bradford Dye Reagent assay (Bio-Rad, Hercules, CA, USA), the whole cell lysate of each sample was subjected to 10% sodium-dodecyl sulphate polyacrilamide gel electrophoresis (SDS-PAGE) together with prestained protein molecular markers sharpmass VII (Euroclone, Milan, Italy). The proteins were electrophoretically transferred onto nitrocellulose membranes Amersham protran 0.2 µm (Cytiva Europe, Freiburg, Germany) for 90 min at 350 mA. Membranes were blocked for 1 h at RT with 10% nonfat milk in Tris Buffered Saline (Bio-Rad) at pH 7.4 containing 20 mM Tris, 500 mM NaCl and supplemented with 0.05% Tween 20 (Bio-Rad), and probed for 1 h at RT with primary antibodies anti-tau (D1M9X), anti-pH2AX and anti-GAPDH (all from Cell Signaling Technology, Danvers, MA, USA), anti-β-Tubulin (Cell Signaling Technology), anti-cyclin β1 (Santa Cruz Biotechnology, CA, USA), anti-beclin 1 (2A4, Cell Signaling Technology) according to dilution suggested by the manufacturer. Protein bands were visualized after 1 h of incubation with horseradish peroxidase (HRP)-conjugated anti-rabbit IgG or anti-mouse IgG (Cell Signaling Technology) at RT, and chemiluminescence reaction, using the ECL Western detection system (Amersham, Buckinghamshire, UK). Chemiluminescent signals were acquired by Chemidoc XRS system and digitally elaborated with Imagelab software (Bio-Rad).

### Knockdown of tau protein by siRNA

Cells were seeded at a density of 5 × 10^4^ cells/mL in a 6 well-plate and in standard culture conditions. Twenty-four hours after seeding, a pool of four different siRNA constructs (Qiagen, Valencia, CA, USA) was diluted in 200 µl culture medium without serum to obtain a final concentration of 20 nM. Ten µl of HiPerFect Transfection Reagent/Qiagen) were added to the diluted siRNA, vortexing. Samples were incubated for 5–10 min at room temperature then added drop-wise onto the cells. The cells were incubated with the transfection complexes under their normal growth conditions and gene silencing was checked after 48 h by western blot. As a negative control, cells were transfected with 20 nM scrambled siRNA (AllStars Negative Control—Qiagen).

### Immunofluorescence

Cells were seeded at a density of 10,000 cells/cm^2^ on glass coverslips pretreated with 30 μg/mL poly-L lysine to promote adherence. At the end of the treatment cells were fixed with 4% paraformaldehyde (Euroclone) for 10 min at 4 °C and permeabilized for 10 min at RT with 0.1% (v/v) Triton X-100 (Bio-Rad). After washing, cells were incubated with anti-tau (D1M9X) anti-pH2AX or anti-β-Tubulin (all from Cell Signaling Technology), for additional 45 min. After washing with PBS, cells were incubated for 30 min at RT with Alexa Fluor 594/488-conjugated secondary antibodies (Jackson ImmunoResearch Laboratories, West Grove, PA, USA). Controls were performed by omitting the primary antibody. Slides were mounted with ProLong Gold antifade mounting medium with DAPI (Cell Signaling Technology). Finally, cells were observed with Zeiss Axio Vert. A1 fluorescence microscope (Zeiss, Jena, Germany) and acquired images were digitally elaborated with a modular image-processing and analysis software (Zen 2012 SP2 Blue Edition).

### Flow cytometry analysis

Flow cytometry was used to analyse the expression of tau and LAMP1 in cancer cells and for the analysis of cell cycle. For protein expression analysis, cells at the endpoint were fixed with 4% paraformaldehyde and permeabilized by 0.1% (v/v) Triton X-100. After washing, cells were incubated with primary antibodies rabbit anti-tau (D1M9X) and anti-LAMP-1 (eBioH4A3, FITC, eBioscience) for 1 h at 4 °C, followed by Alexa Fluor 594-conjugated anti-rabbit antibody (Jackson ImmunoResearch Laboratories, West Grove, PA, USA) for additional 30 min. All samples were analysed with BD Accuri C6 Flow cytometer (BD Technologies, Durham, NC, USA) equipped with a blue laser (488 nm) and red laser (640 nm). At least 20,000 events were acquired for each experimental point. Cell cycle analysis by cytometer was performed following standard staining protocol. Briefly, at the endpoint cells were washed twice in phosphate buffered saline (PBS) and fixed in 70% ethanol for 10 min at 4 °C. Then, cells were washed twice with PBS, resuspended in 0.5 mL PBS, 50 μL RNase A (5 μg/mL) (Sigma-Aldrich), and stained with 0.5 mL of 100 mg/mL propidium iodide (Sigma-Aldrich) in PBS. Cells were incubated for 30 min at room temperature in the dark and analysed for DNA content and the fluorescence intensity was measured using the Accuri C6 cytometer.

### Data analysis and statistics

All the statistical procedures were performed by GraphPad Prism Software Inc. (San Diego, CA, USA). Data are expressed as means ± standard deviations (SDs) of at least three independent experiments. The statistical significance between measure series was calculated with parametric Student *t* test and *p* values of less than 0.05.

## Results

### Autophagy protects from docetaxel cytotoxicity

**Fig. 1:**
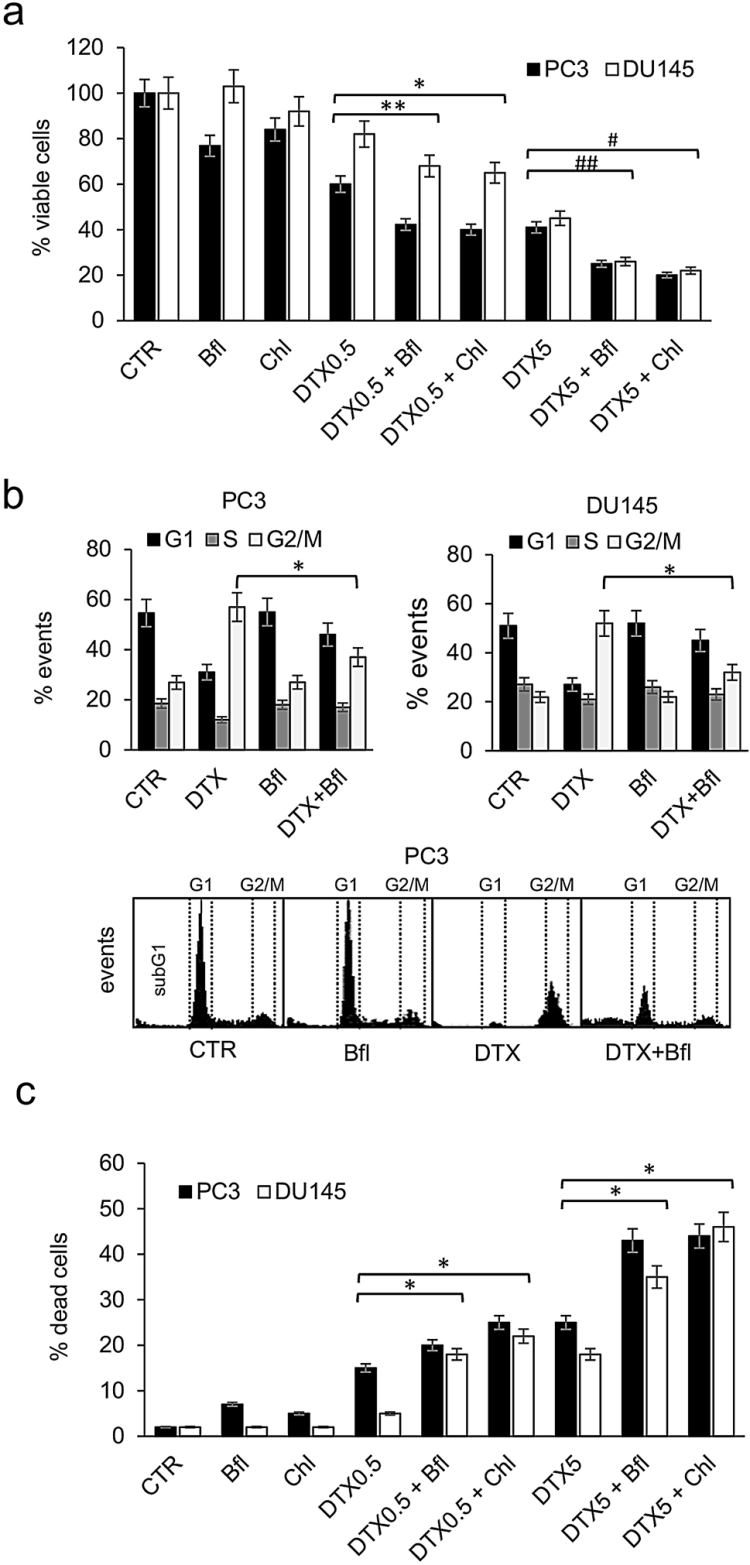
Analysis of combination treatment with docetaxel and autophagy inhibitors. **a** PC3 and DU145 cells were treated for 72 h with two concentrations of docetaxel (DTX, 0.5 and 5 nM) alone or in combination with two different autophagy inhibitors, bafilomycin (Bfl 1 µM) and chloroquine (Chl 20 µM); the number of viable cells were measured and the mean of five different measures with SDs was reported in the histogram. The differences between DTX alone and combination treatments resulted statistically significant (Student’s *t* test, *p* < 0.05) and are indicated in the histogram with symbols: (*) DTX0.5 + Chl vs DTX0.5; (**) DTX0.5 + Bfl vs DTX0.5; (#) DTX5 + Chl vs DTX5; (##) DTX5 + Bfl vs DTX5. **b** PCa cells were treated for 72 h with docetaxel alone (DTX, 0.5 nM) or in combination with bafilomycin (Bfl 1 µM) and the number of events in cell cycle phases was measured by cytofluorimetry. The experiment was repeated three times and means plus SDs were reported in the histogram, while representative profiles obtained from PC3 cells analysis are shown in the bottom panel. Asterisk indicates a value *p* < 0.05 (Student’s *t* test) between number of cells in G2/M phase between DTX + Bfl and DTX alone. **c** After 72 h of treatment performed as described in panel (**a**), PC3 and DU145 cells were evaluated for the presence of dead cells. The histogram shows the means and SDs from five different experiments and the statistically significant differences between DTX alone and combination treatments are indicated by asterisks (Student’s *t* test, *p* < 0.05)

To evaluate the potential interaction between docetaxel-induced cytotoxicity and autophagy, PCa cell lines, PC3 and DU145, were treated with bafilomycin A1 (Bfl) or chloroquine (Chl) in combination with different concentrations of docetaxel (0.5 and 5 nM). Bfl and Chl are common and potent inhibitors of cellular autophagy interfering in the formation of autolysosome (Vinod et al. 2014; Maclean et al. 2008). When viable cells were counted after 72 h of treatment, a significant decrement in the number of cells were recorded in presence of the combination docetaxel plus autophagy inhibitor respect to all other experimental conditions (Fig. 1a). This effect was observed both in PC3 and DU145 cells and demonstrated a similar trend for bafilomycin and chloroquine. The analysis of the cell cycle by cytofluorimetry revealed that the antitumoral effect of the combination was not associated with an increased number of cells blocked in G2/M phase of the cell cycle, in fact the addition of bafilomycin and chloroquine to docetaxel stimulated a reduction in the number of cell in G2/M phase respect to docetaxel alone (Fig. 1b). At the same time, the measurement of the number of dead cells revealed that the increased antitumoral efficacy of the combination treatment was dependent on a higher capacity to induce cell death (Fig. 1c).

**Fig. 2:**
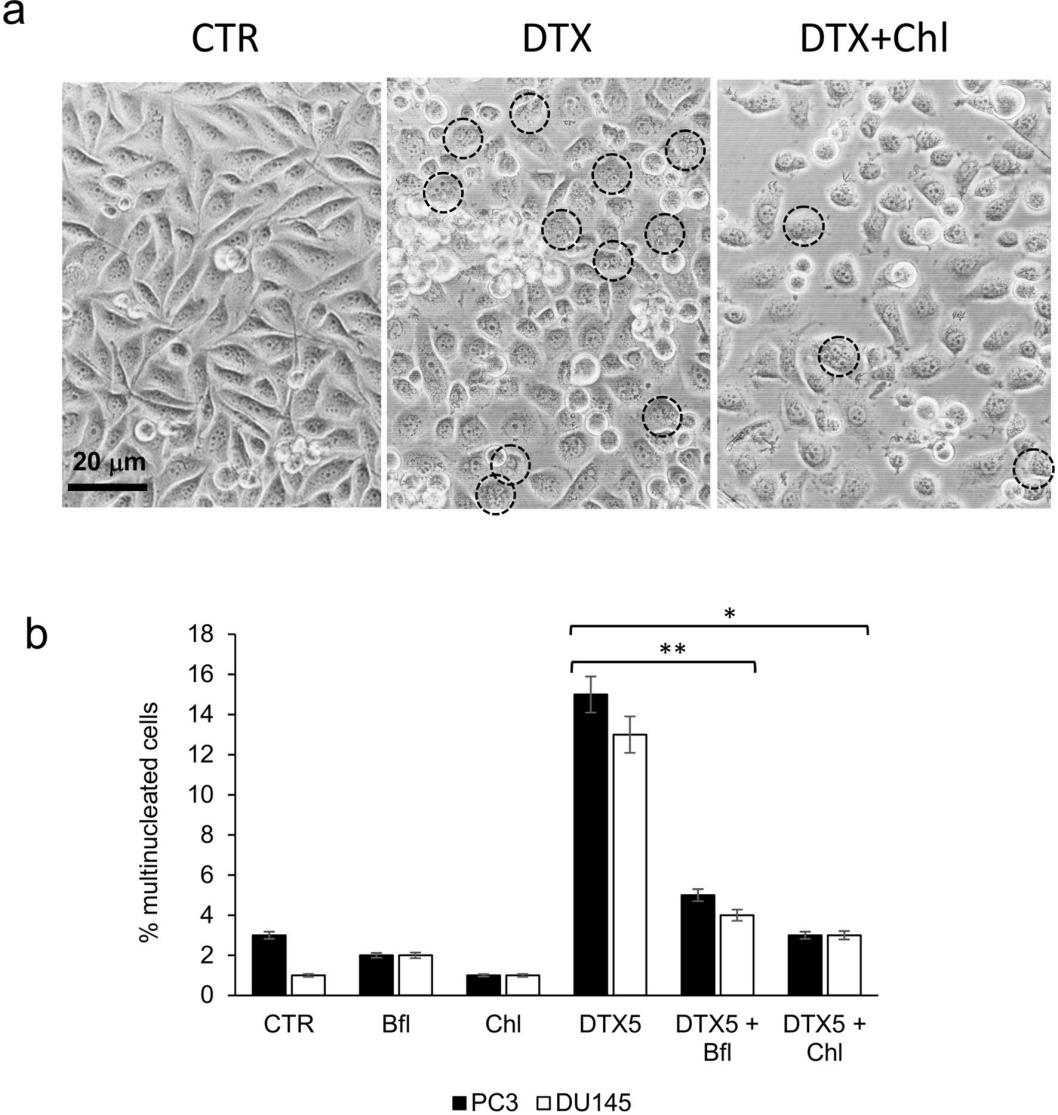
PC3 and DU145 cells were treated for 72 h with 5 nM docetaxel (DTX) alone or in combination with bafilomycin (Bfl 1 µM) or chloroquine (20 µM), and then cells were observed with phase-contrast microscopy for the presence of multinucleated cells. **a** Exemplificative images of PC3 cells treated with DTX and chloroquine (Chl), in which multinucleated cells were evidenced by black circles. **b** The mean number of multinucleated cells for each experimental point was counted evaluating ten different optical fields with 40×magnification, and it was reported with SDs in the histogram. Asterisks indicate a statistically significant difference (Student’s *t* test, *p* < 0.05) between number of multinucleated cells: (*) DTX5 + Chl vs DTX5; (**) DTX5 + Bfl vs DTX5

It is well known the capacity of docetaxel to induce the arrest in G2/M phase of the cell cycle and the concomitant emergence of multinucleated cells (Mittal et al. 2017). We confirmed this phenomenon in PC3 and DU145 cell lines and the appearance of multinucleated cells was well visible also by phase-contrast microscopy (Fig. 2a). After 72 h of treatment with 5 µM docetaxel the percentage of multinucleated cells increased to about 15% and 13% in PC3 and DU145, respectively. The addition of bafilomycin or chloroquine in docetaxel-treated cells counteracted significantly the formation of multinucleated cells, that were lower than 5% of the total number of cells (Fig. 2b).

### Inhibition of autophagy determines accumulation of tau oligomers

**Fig. 3:**
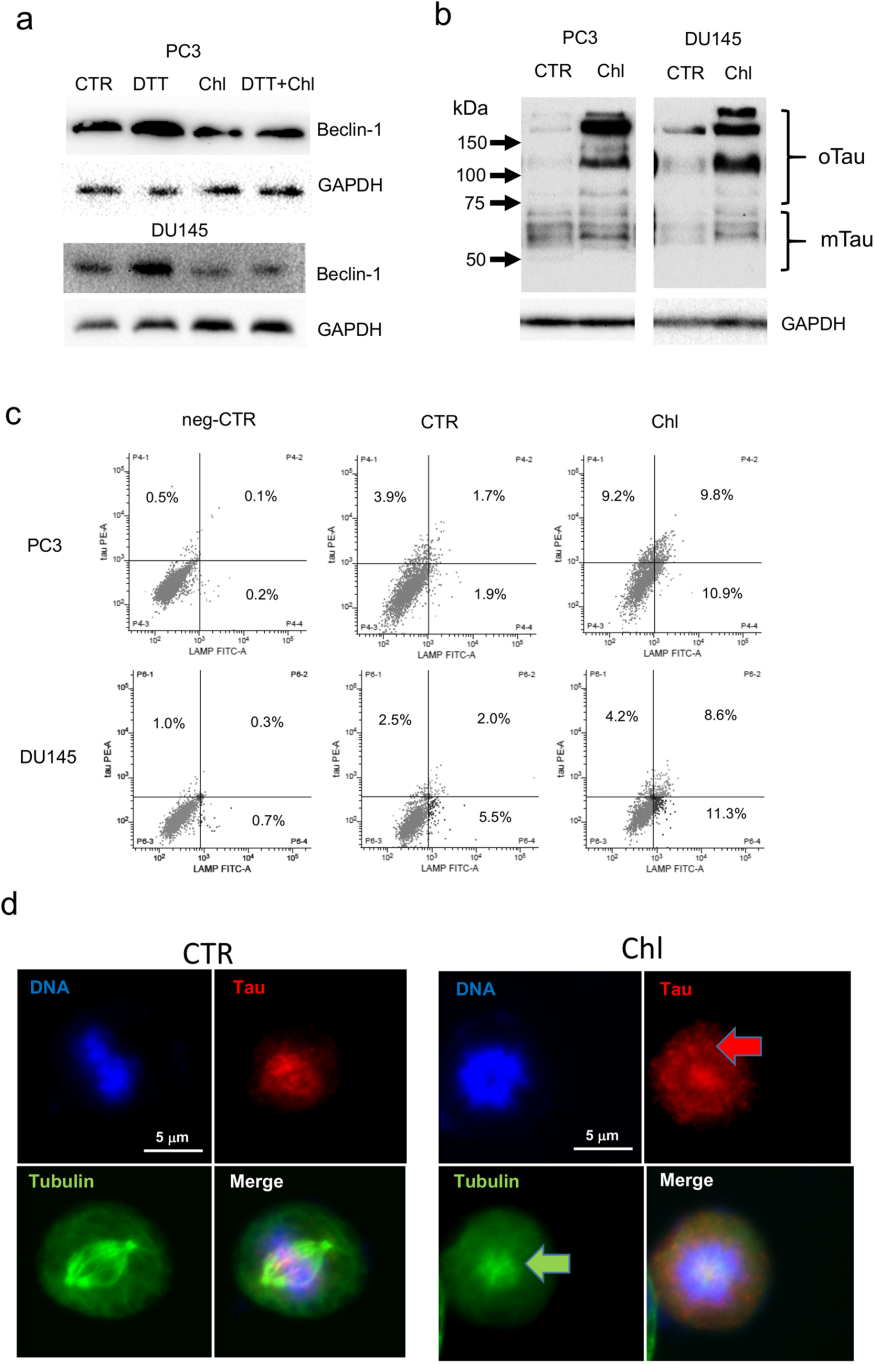
**a** Beclin1 expression was analysed by western blot in PC3 and DU145 cells after 48 h treatment with 0.5 mM DTT, 20 µM chloroquine (Chl), and combination (DTT + Chl). **b** The expression of tau was evaluated by western blot of total cell lysates from PC3 and DU145 cells treated with 20 µM chloroquine for 72 h. The complete pattern of immunodetection was shown with the indication of oligomeric (oTau) and monomeric tau forms (mTau) evinced by molecular weight. Expression of GAPDH from the same cell lysates was shown as a loading control. **c** Flow cytometry analysis of PC3 (upper panels) and DU145 (bottom panels) cells treated with 20 µM chloroquine for 48 h and immunostained with anti-LAMP1 (FITC) and anti-tau (PE). The left panels show the fluorescence pattern of negative control cells (background fluorescence), and percentages of positive cells are indicated within each quadrant. **d** Tau expression was evaluated by immunofluorescence in PCa cells treated with 20 µM chloroquine for 72 h and it was correlated with the pattern of DNA, stained with DAPI, and of immunodetected β-tubulin. Exemplificative images from control and chloroquine-treated PC3 cells showing in dividing cells the expression pattern of DNA (blue), tau (red) and β-tubulin (green). The fourth image for each series represents the digital combination (merge) of the previous three images taken by immunofluorescence microscopy. The arrows indicate the peculiar pattern of tau (red arrow) and β-tubulin (monoastral spindle, green arrow) in chloroquine-treated cells respect to control cells

DU145 cells are considered autophagy-resistant cells in some experimental conditions because they do not express ATG5. For this reason, we verified in these cells if autophagy was induced by endoplasmic-reticulum (ER) stress conditions. We treated PC3 and DU145 with 0.5 mM DTT, a well-known ER-stress inducer, and verified the expression of beclin-1 (Fig. 3a). DTT treatment for 48 h determined the upregulation of beclin-1 in both cell lines, while chloroquine was able to counteract this increment. In addition, the analysis of total cell lysates from PC3 and DU145 cells treated with chloroquine revealed a shift in the relative abundance of different forms of tau protein. The western blot analysis permitted to detect several forms of tau protein that migrated with different molecular weights. According to the literature we can recognize as lower molecular weight forms (below 70 kDa) different isoforms due to alternative splicing and post-translational modification, whereas the higher molecular weight forms, above 70 kDa, are represented by soluble oligomers. The treatment with chloroquine determined an evident accumulation of tau protein, mainly as high-molecular weight oligomers in both PC3 and DU145 cell lines (Fig. 3b). To verify the concomitant accumulation of autophagy vesicles and tau protein we performed a double fluorescence immunostaining with antibodies anti-LAMP1 and anti-tau in cells treated with chloroquine. The cells were analysed by flow cytometry and results showed that 24 h chloroquine treatment determined the up-regulation of LAMP1 and tau positive cells respect to untreated cells and that more than 50% of tau positive cells were also positive for LAMP1 (Fig. 3c). The analysis of cells by fluorescence microscopy for tau expression revealed a peculiar distribution of the positivity: in control cells the expression of tau was barely observable in the G1-phase cells, while tau become evident in M-phase cells with a clear association with the mitotic spindle (Fig. 3d). In cells treated with chloroquine we confirmed the positivity of cells in mitosis but in some cases with a different pattern of staining. In fact, tau was evident not only on the mitotic spindle but also in the cytoplasm. The concomitant analysis of β-tubulin in these cells revealed frequently an aberrant phenotype, e.g., monoastral spindle, associated with non-aligned chromosomes (Fig. 3d). This phenotype was interpreted as a consequence of any interference with the formation of the mitotic spindle.

### Formation of tau oligomers is associated with DNA damage and sensitization to docetaxel

**Fig. 4:**
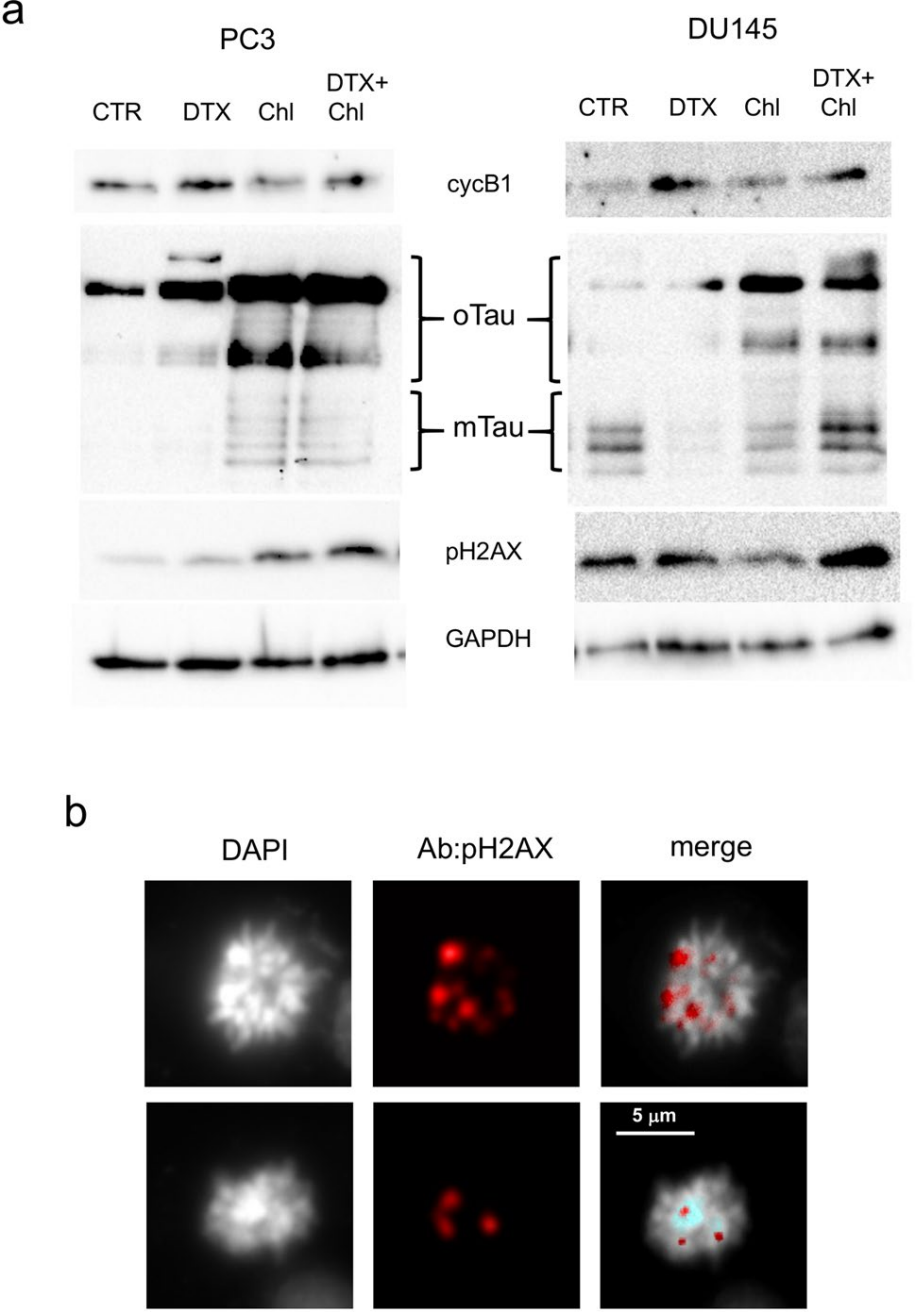
Analysis of pH2AX expression in PCa cells treated with docetaxel and autophagy inhibitors. **a** PC3 and DU145 cells were treated for 72 h with 5 nM docetaxel alone or in combination with 20 µM chloroquine, and then total lysates were analysed by western blot for the expression of selected markers: cyclin B1 (cycB1, marker of G2/M cell cycle phase), tau (with the indication of oligomers and monomers) and pH2AX (marker of DNA DSB). The expression of GAPDH was evaluated as loading control. **b** Sample images of aberrant mitotic PC3 cells treated with 5 nM docetaxel in combination with 20 µM chloroquine showing DNA content stained with DAPI (white) and pattern of immunodetected pH2AX (red). A digital combination (merge) of the two previous images is shown to visualize the localization of DSB on DNA

**Fig. 5:**
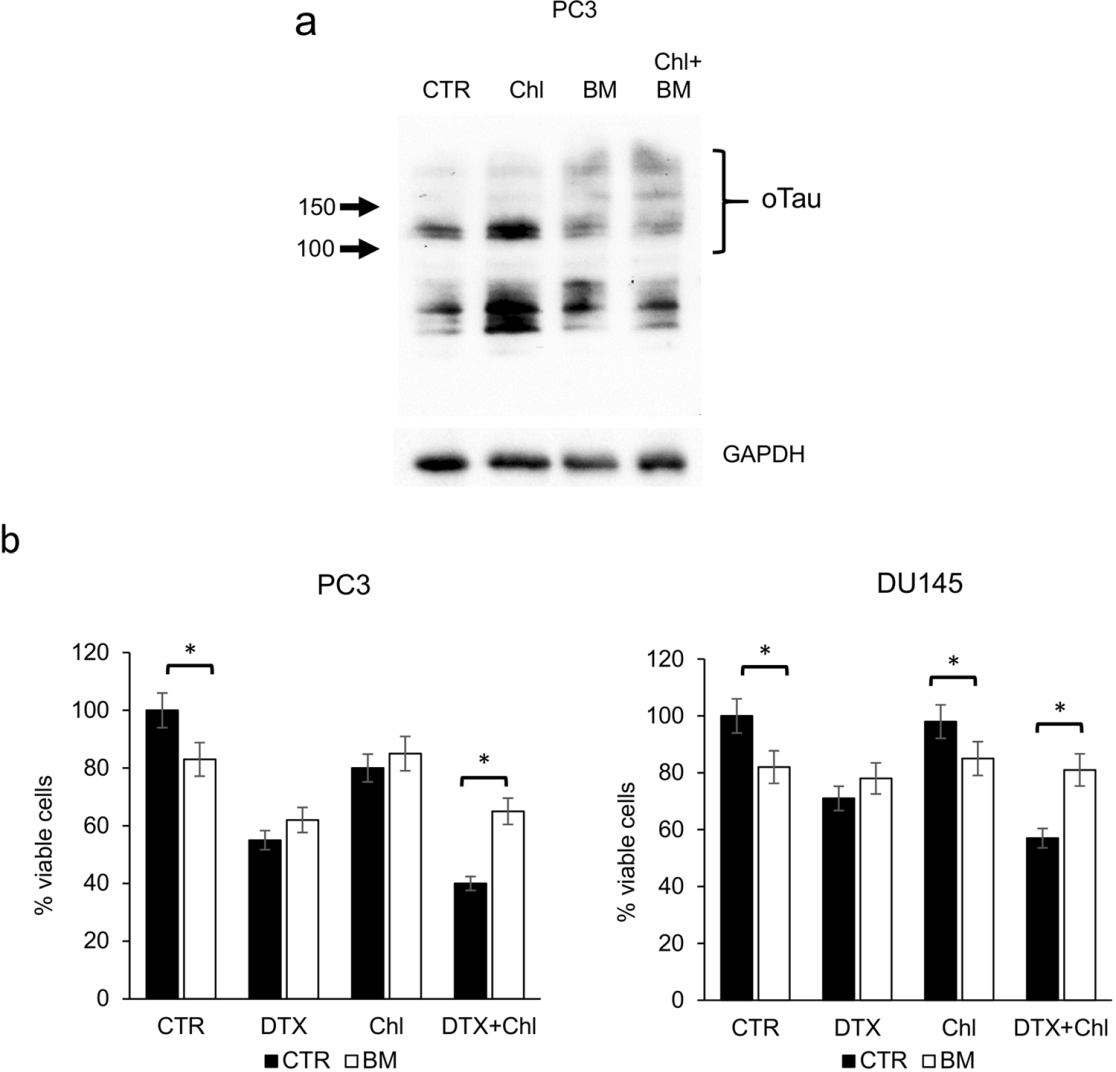
Effect of methylene blue (BM) in docetaxel and combination-treated PCa cells. **a** The expression of tau oligomers was evaluated by western blot of total lysates from PC3 cells treated with 20 µM chloroquine alone or in combination with 10 µM BM for 72 h. The complete pattern of immunodetection was shown with the indication of oligomeric (oTau) and monomeric tau forms (mTau) evinced by molecular weight. Expression of GAPDH from the same cell lysates was shown as a loading control. **b** PC3 and DU145 cells were treated for 72 h with 5 nM docetaxel alone or in combination with 20 µM chloroquine (black bar); the same experimental series were performed in presence of 10 µM BM (white bar); the number of viable cells was measured and the means of five different experiments with SDs are reported in the histogram. When present, the statistical significant differences (Student’s *t* test, *p* < 0.05) between BM-treated and respective BM-untreated cells are indicated in the graph by asterisk

**Fig. 6:**
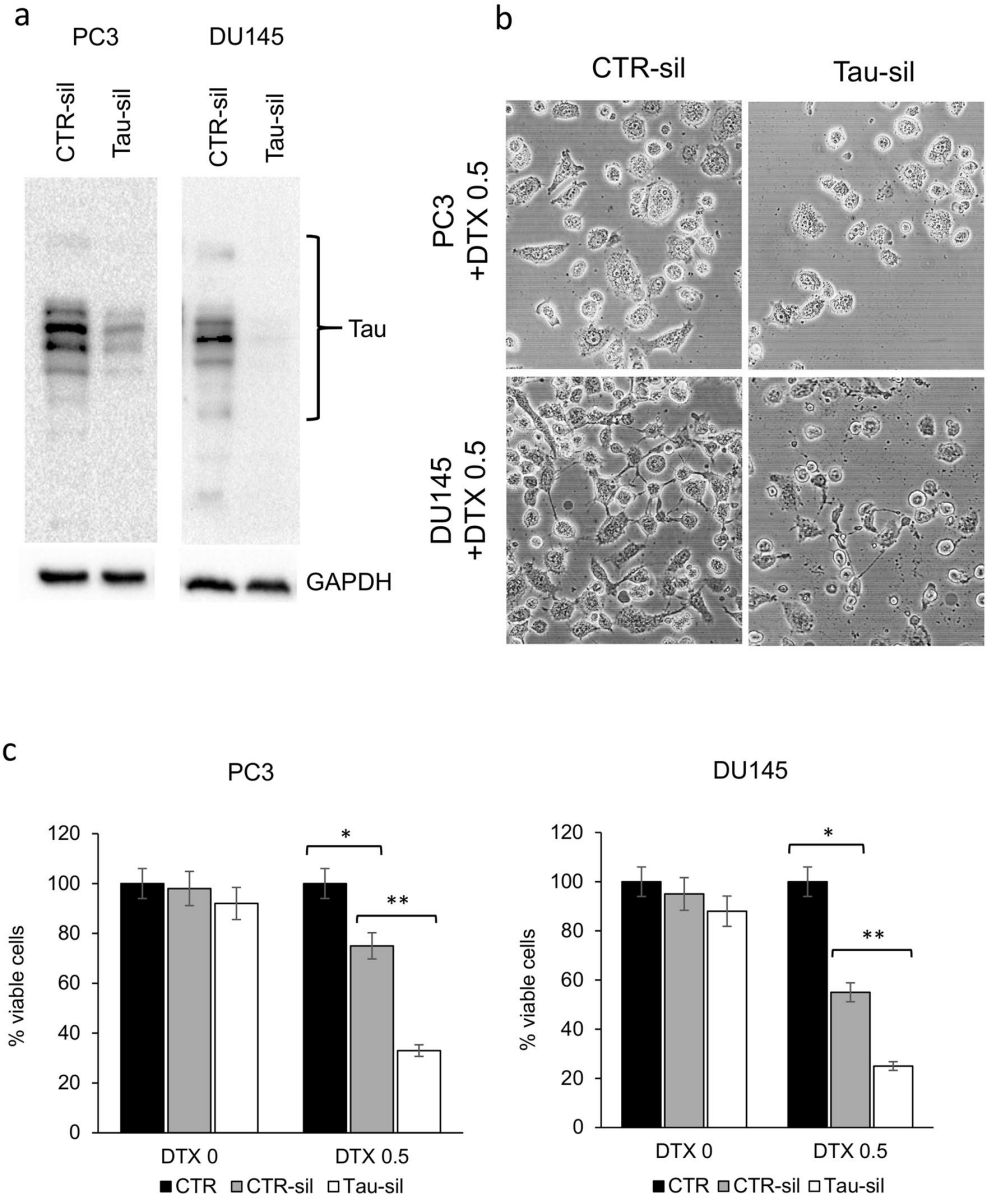
Short-term Tau silencing was obtained in prostate cancer cells by siRNA interfering method. **a** The expression of tau was evaluated by western blot of total lysates collected from PC3 (left) and DU145 (right) 48 h after transfection with tau-targeted siRNA (tau-sil) or control random siRNA (CTR-sil). The expression of GAPDH was evaluated as loading control. **b** Tau-silenced and CTR-silenced PC3 and DU145 cells were treated for 48 h with 0.5 nM docetaxel (DTX) and then cells were observed with phase-contrast microscopy for the presence of multinucleated giant cells. Exemplificative images of tau-silenced and CTR-silenced cells treated with DTX are shown. **c** The same experimental series were analysed for the presence of viable cells, and the means of five different experiments with SDs are reported in the histogram. CTR indicates untreated cells. The presence of statistical significant differences (Student’s *t* test, *p* < 0.05) are indicated in the graph by asterisk: (*) CTR vs DTX-treated CTR-silenced cells; (**) DTX-treated CTR-silenced vs DTX-treated tau-silenced cells

Then we evaluated whether the formation of the aberrant mitotic spindle and the inhibition of correct chromosome separation was associated with DNA damage. We treated PCa cells with docetaxel, chloroquine or their combination, and then analysed the expression of selected proteins, including cyclin b1, tau and pH2AX. The increase in cyclin b1 confirmed the G2/M block associated with docetaxel treatment (Fig. 4a). As expected, tau oligomers were particularly evident when cells were treated with chloroquine, and analysis of lysate from combination treatment showed that tau accumulation was stimulated by chloroquine also in presence of docetaxel. When we analysed pH2AX, which expression is associated with DNA double-strand breaks (DSBs), we observed an increase in the combination treatment, respect to single treatments and control. To localize on cells the expression of pH2AX, we performed an immunofluorescence analysis that revealed the association of pH2AX with not segregated chromosomes in cells showing the aberrant mitotic spindle (Fig. 4b). To evaluate the role of tau oligomers in cooperation with docetaxel-induced cytotoxicity we chose a compound, methylene blue (BM), that was described as able to counteract the formation of tau aggregates in vitro and in vivo (Hosokawa et al. (2012)). We first confirmed by western blot analysis that BM inhibited the formation of tau oligomers induced by chloroquine (Fig. 5a). Then we added BM as pre-treatment, to PC3 and DU145 cells treated with docetaxel, chloroquine or their combination, and evaluated cell viability after 72 h. BM was able to significantly revert the cytotoxicity increase due to the addition of chloroquine to docetaxel (Fig. 5b). Because BM exerted its effect without affecting in a significant manner the expression of monomeric tau forms, we aimed also to verify the possible role of these forms in the sensitivity to docetaxel. By siRNA-based interference method, tau expression was transiently silenced in cancer cells, reducing after 48 h the protein expression of more than 80% respect to control cells (Fig. 6a). When tau-silenced cells were treated with docetaxel we observed after 48 h a reduction in the formation of giant hyperploid cells respect to control cells treated with the same docetaxel concentration (Fig. 6b). This phenomenon was paralleled by a significant reduction also in the viability of tau-silenced cells treated with docetaxel respect to control cells treated with docetaxel (Fig. 6c).

## Discussion

Soluble tau oligomers of differing sizes have been reported in several studies. Using recombinant protein, dimers and trimers were observed in SDS-PAGE with apparent size greater than 100 kDa (Patterson et al. 2011; Makrides et al. 2003; Lasagna-Reeves et al. 2010). These oligomers can be seeds for larger aggregates containing six to eight tau molecules (Sahara et al. 2007). The oligomers themselves have been shown to be toxic both in vitro and in vivo (Lasagna-Reeves et al. 2010, 2011). In addition, in neuroblastoma cell line SH-SY5Y, oligomers demonstrated a higher cytotoxic capacity with respect to tau monomers or filaments. Tau hyperphosphorylation and misfolding are the earliest disease-associated changes observed in tauopathies. Although many studies have demonstrated that dysregulation in tau phosphorylation is a common cause for the accumulation of pathologic tau oligomers (Alonso et al. 2001), other data indicate that tau aggregation was also phosphorylation-independent and that the formation of tau oligomers and/or complexes with other proteins is determined by other post-translational modifications or simply by an excessive free cytosolic tau (Goedert et al. 1996). This phenomenon is confirmed by the evidence that tauopathies can be mimicked in many different models simply through the genetic hyperexpression or the addition of exogenous tau protein.

The mechanism underlying the toxic effect of tau oligomers is currently unknown, however available data and our results permit to speculate about a possible interference in the formation of the mitotic spindle. Indeed, since the observation of mitotic defects in tauopathies, association between tau and mitosis has represented an intriguing field of studies also in non-neuronal cells (Herrup 2010). Examples of dysfunction in microtubule association derived mainly from studies about specific mutated forms of tau. Mutations that compromised microtubules binding capacity or their assembly have been associated with aberrant mitotic spindle and chromosome missegregation. The initial observation of the presence of aneuploidy in peripheral cells of patients affected by neurodegeneration due to dominant mutation in tau seems to confirm in vivo a role of tau in genome stability (Rossi et al. 2008). In addition, there is a growing literature on aberrant cell cycle activation and re‐entry, DNA damages and checkpoint activation in tauopathies (Khurana et al. 2012). Aberrant mitosis is also induced by WT tau, and in particular in association with overexpression/accumulation. It has been reported that 4R-Tau isoform overexpression in Drosophila wing discs induced the formation of monopolar spindles leading to aneuploidy and cell death also in non-neuronal cells (Bougé and Parmentier 2016). Similar phenomena were described by other authors in the development of Drosophila larval brains, with an increased level of aneuploidy. Importantly the same authors described the formation of monopolar spindles when WT or mutant tau were overexpressed in neuroblastoma cell line SH-SY5Y (Malmanche et al. 2017). Our data confirmed that accumulation of tau in the cytoplasm of mitotic cells is associated with the formation of monopolar spindles, both in control and docetaxel treated cells and this suggests the existence of a specific molecular mechanism interfering with the formation of the mitotic spindle.

Although tau overexpression, and in particular of high molecular weight isoforms, seems to be sufficient to induce mitotic defects, current knowledge does not permit to precisely describe the mechanism determining the antimitotic effect of tau. Tau is known to be hyperphosphorylated during mitosis and to localize to the mitotic spindle (Connolly et al. 1977). An interaction between 4R-Tau and the motor kinesin Kip69F (kinesin-5) during mitosis was described. Importantly Bougè and Parmentier demonstrated that excess of human tau protein inhibited the Eg5 kinesin and cell mitosis in Drosophila (Bougé and Parmentier 2016). In addition, imaging studies of kinesin motor proteins, kinesin 1 and Eg5, moving along microtubules showed that the binding of tau tended to block their motor activity. In particular, an alteration in tau availability can compromise the proper organization and stability of spindle microtubules (Dixit et al. 2008; Ma et al. 2011). The microtubule-binding protein MAP4, paralog of tau, has a similar negative effect on mitosis, and when transiently expressed in different human haematological cancers, MAP4 induced mitotic arrest with the presence of monopolar spindles (Holmfeldt et al. 2003). The fact that antimitotic effect of tau was only sporadically reported when transfected in cells could be due to the efficient clearance mechanism or alternative pathways. Indeed, in our experiments the most visible cytotoxic effect of tau oligomers was revealed with the concomitant inhibition of autophagy and the presence of mitotic stress. Otherwise, in basal conditions, we observed only a few aberrant mitotic figures, associated probably to phenotypic cancer heterogeneity. In agreement, Li et al. observed a slight but significant increase in G2/M phase in HEK293 cells transfected with the 1N3R Tau isoform (Li et al. 2015).

Another hypothesis is that tau soluble oligomers can interact and change the localization of other proteins involved in mitosis. In fact, tau has been shown to localize in many intracellular districts other than cytoskeleton and there is evidence to support the hypothesis that post-translational modifications can modulate its localization and function. For example, tau interacts with several kinases and phosphatases that have oncogenic potential (Mandelkow and Mandelkow 2012). Several ribonucleoprotein complexes (RNPs) can form toxic granules containing tau (Maziuk et al. 2017) and the overexpression of exogenous tau oligomers is sufficient to induce changes in localization and function of Musashi1 RBP in a dose dependent-manner (Montalbano et al. 2019). Also, tau may shuttle between cytoplasm and nucleus, as canonical heat-shock proteins do when they induce the stress response and, interacting with RNA and DNA, it can protect the neuron from senescence (Ulrich et al. 2018).

In addition, tau could also interact directly with chromosomes and protect them during mitotic segregation. Tau was detectable in nucleus and its presence contributes to maintain DNA double helix structure and DNA winding (Greenwood and Johnson 1995; Hua and He 2003; Frost et al. 2014). The in vivo genetic ablation of tau determined the accumulation of DNA breaks in specific DNA regions and in particular in pericentromeric chromatin (Mansuroglu et al. 2016). More than a structural protein, tau is considered a guardian of DNA, playing a role during cellular stress, when it was shown to translocate to the nucleus, preventing stress-induced DNA breaks (Sultan et al. 2011). The protective function by tau was well described under mild oxidative stress conditions, whereas pathological oxidative stress conditions lead to the formation of toxic tau oligomers. Interestingly, the same authors noted that hyperthermia induced a strong increase in γ-H2AX foci selectively in KO-tau neurons suggesting a direct role of tau in the DNA DSB repair in vivo (Violet et al. 2014; Violet et al. 2015). In our experiments we observed that the concomitant inhibition of autophagy and docetaxel treatment stimulate the formation of DNA DSBs, suggesting a direct association between DNA integrity and tau accumulation. Stimulatingly, DNA DSBs were evident in aberrant mitotic figures, and thus this renders plausible that DNA damage is a consequence of the toxic effect of tau oligomers on the formation of mitotic spindle rather than a direct effect on DNA integrity. These studies clearly highlighted the existence of critical time frames, after stress injury or during mitosis, during which monomeric tau can play a nucleic acid protective function. Thus, events triggering tau oligomerization, with the loss of the monomeric form, could render more vulnerable DNA to stress-induced damages. Oxidative stress is a basal characteristic of many cancers, and also an inducer of tau oligomerization, therefore protective mechanisms able to remove tau oligomers, such as autophagy, are fundamental in assuring cancer cell survival.

Cancer is frequently associated with chromosome aberrations, including polyploidy and aneuploidy, thus it is questionable whether tau could play a role in carcinogenesis or tumor progression (Giam and Rancati 2015). Although cancer cells show resistance to polyploidy, the continuous mitotic failure can induce programmed cell death, necrosis or senescence (Vitale et al. 2011a). A probable event seen in cancer cells in presence of unrepairable genomic instability is mitotic catastrophe (MC). Although the high degree of variability in MC does not permit to precisely characterize the molecular pathways involved, the leading event associated with this onco-suppressive event is the interference with the spindle assembly checkpoint that controls proper attachment of the chromosomes to the spindle microtubules, thus stimulating impaired segregation of genetic material between daughter cells, chromosomal breaks, clumps of chromatin and deficient karyokinesis (Castedo et al. 2004; Vitale et al. 2011b). The reduced number of multinucleated cells we observed in presence of autophagy inhibitors respect to docetaxel alone suggests that a fast permanence in M phase could be a protective mechanism against docetaxel toxicity. It is plausible that tau can protect from MC sustaining correct formation of mitotic spindle and segregation of chromosomes, allowing the bypass of the spindle assembly checkpoint. Microtubule inhibitors such as taxanes reduce dynamic turnover of mitotic spindle and activate spindle mitotic checkpoint, leading preferentially to MC. However, a possible outcome is also mitotic slippage in which occasionally cells could continue dividing without cytokinesis. Although the mechanisms that determine the fate of individual cells cannot be predetermined, it is plausible that molecular events that facilitate the exit from mitotic arrest are favourable for cancer survival. In this sense Inoue et al. suggested that autophagy may contribute to sensitization to chemotherapy by turning off spindle mitotic checkpoint and by shifting MC (Inoue et al. 2014).

Increased autophagy is a hallmark of advanced malignancies and it is supposed to confer a selective advantage during aberrant cell proliferation and in adverse microenvironmental conditions (Galluzzi et al. 2015). Autophagy can act as either pro-death or pro-survival factor, depending on the stage of cancer and microenvironmental context considered, and this paradoxical aspect is evident also during therapeutic intervention. In fact, autophagy may enhance cancer therapy efficacy through the direct or indirect promotion of cell death, but it can also enhance chemoresistance (Chen et al. 2010). Although in mammalian cells autophagy has been implicated in the turnover of cytoplasmic content, involvement in homeostasis of nuclear components has also been observed. Autophagosomes containing nuclear components were observed in wild-type cells, and more frequently in cells with nuclear defects, including envelopathies and improper chromosome segregation (Park et al. 2009; Rello-Varona et al. 2012). Autophagy is also important for the timely removal of aggregated forms of pathogenic proteins, including tau. The clearance of misfolded proteins is mainly mediated by chaperone-mediated autophagy (CMA) and macroautophagy. Autophagic inhibition by 3-methylamphetamine or chloroquine was shown to counteract tau clearance, leading to tau aggregation (Hamano et al. 2008). On the other hand, stimuli that activate autophagy, including rapamycin, was shown to inhibit the accumulation of tau aggregates in vivo (Rodriguez-Navarro and Cuervo 2010). In addition, autophagy has been shown to play a key role in sustaining cell survival during mitotic arrest and MC (Inoue et al. 2014; Maskey et al. 2013). Our data suggest that inhibition of autophagy in prostate cancer cells determine a little reduction of proliferation, significant in PC3 cells, in basal conditions, and a significant additive cytotoxic effect when used in combination with docetaxel. Because docetaxel induced an intense block in G2/M phase of the cell cycle in our cell model, it is plausible that autophagy exerts a protective effect just during cell division. It is unclear how autophagy can play a protective role during mitotic arrest, however, there is evidence of p53-dependent mechanism able to stimulate cell death in parallel with inhibition of autophagy (Xiao et al. 2015). Significantly, both cell lines we used in our study express non-functional p53, allowing autophagy to function also in presence of docetaxel-induced DSBs, and accounting for a partial resistance to the therapy.

The most consolidated evidence for tau involvement in cancer progression is currently represented by the predictive value of tau expression in breast and ovarian cancer. In prostate cancer, few data are available about a possible interaction between tau and taxanes treatment. Yang et al. demonstrated in the same cell lines used by us that inactivation of tau sensitized cancer cells to docetaxel toxicity. Interestingly these authors noted an upregulation of PI3K/Akt/mTOR pathway in docetaxel resistant cells, suggesting a possible involvement of autophagy in the drug resistance (Yang et al. 2017). Other authors have confirmed the important role of autophagy as modulator of chemotherapy in prostate cancer, but also showing the dependence of this phenomenon on the cell model used (Cristofani et al. 2018). In fact, the mechanism underlying this clinically relevant aspect is largely unexplored. We propose that autophagy can counteract docetaxel toxicity modulating the expression level of tau protein. Some authors have previously linked resistance to taxanes to competitive action with tau for the same tubulin-binding domain, proposing an antagonizing role for microtubule stabilization (Kar et al. 2003). At the same time silencing of tau led to a significant decrease of survival ability of carcinoma cells treated with paclitaxel (Gurler et al. 2015). We confirmed these results and propose that tau oligomer-associated toxicity is realized through the interference with the protective role of tau monomer during mitosis. In fact, our data seem to indicate that the cooperative role of tau oligomers with docetaxel-induced cytotoxicity could be mimicked by silencing tau expression. Thus, our hypothesis is that the accumulation of tau oligomers, that we reached through autophagy inhibition, determined the sequestration of tau monomers interfering in their physiological activity and promoting cancer cell death.

In conclusion, we confirm the importance of tau homeostasis in docetaxel sensitivity and extend this view proposing that not only the presence of tau can protect from docetaxel cytotoxicity but that accumulation of tau can cooperate with the antimitotic drug. We speculate that the overexpression of tau in cancer could confer a selective advantage, mainly in the stabilization of DNA and/or mitotic spindle, in cancer cells characterized by aberrant highly heterogeneous genetic background. However, the peculiar biology of tau, that implicates the equilibrium with oligomeric forms, whose activity seems prevalently cytotoxic, could represent an Achilles heel for the cancer cell. In fact, to avoid the formation of progressively aggregating toxic tau oligomers, cancer must maintain a high protein turnover, mainly during mitosis, through the activation of autophagic removal. According to this hypothesis the inhibition of autophagy, interfering with tau turnover, could represent an effective strategy to counteract the resistance to antimitotic agents.
